# Anorectal Malformations (Part 3)

**Published:** 2015-07-01

**Authors:** Sushmita Bhatnagar

**Affiliations:** Department of Pediatric Surgery, B.J.Wadia Hospital for Children, Mumbai

 (This section is meant for residents to check their understanding regarding a particular topic)

## Questions

1. What is long-term outcome for children with ARM?
2. What is Normal Continence mechanism?
3. What is assessment of fecal incontinence (FI)?
4. What is management of fecal incontinence?

## Answers

**Answer 1**

The aim of treatment of anorectal malformations is not just to create a passage for stools in the perineum but also to have a child who can have voluntary bowel movements without any medications and without any associated iatrogenic or congenital abnormality such as urinary incontinence. An assessment and appropriate management of urinary system pathologies (1)is an important aspect of management of a child with anorectal malformations and has been enlisted as one of the criteria for long term assessment by few researchers. (2-6) The quality of life of a child with anorectal malformation is thus dependent on the following factors:

1. Fecal continence

2. Constipation 

3. Urinary continence/Urinary pathologies

The global assessment of long-term outcome of children with various types of anorectal malformations as analysed by Lewitt, et al (7) are tabulated in Table 1. 

**Figure:**
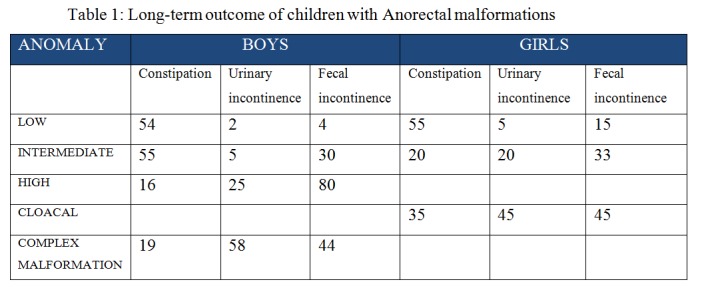
Table 1: Long-term outcome of children with Anorectal malformations

The terminologies used in the outcome analysis are constipation, urinary incontinence and fecal incontinence and must be clearly understood by the students and the researcher before categorizing the patients. 

**Constipation:**

Definition: The North American Society of Gastroenterology, Hepatology, and Nutrition (NASPGHAN) defines constipation as "a delay or difficulty in defecation, present for 2 weeks or more, and sufficient to cause significant distress to the patient." (8)

The Paris Consensus on Childhood Constipation Terminology (PACCT) defines constipation as "a period of 8 weeks with at least 2 of the following symptoms: defecation frequency less than 3 times per week, fecal incontinence frequency greater than once per week, passage of large stools that clog the toilet, palpable abdominal or rectal fecal mass, stool withholding behavior, or painful defecation." (9)

Lewitt and Pena have graded constipation in children with anorectal malformations as follows:

N = Normal (no constipation)

0 = managed with diet restrictions only

1 = managed with laxatives

2 = managed with enemas

3 = severe; not manageable

**Fecal incontinence:**

Definition: An inability to hold feces in the rectum due to failure of voluntary control over the anal sphincters permitting untimely passage of feces and gas is defined as fecal incontinence. 

In a child with anorectal malformation, total continence is only when there is voluntary bowel movement and no soiling. Those children who remain clean/dry on regular bowel management program are pseudo continent. 

**Grades of fecal incontinence:**

A. Voluntary bowel movements or involuntary escape of feces
B. Soiling
a. Normal: No soiling
b. 1 = minimal, occasional, less than 2 times a week; no change of underwear required
c. 2 = frequent; once a day; frequently requires change of underwear
d. 3 = constant

**Urinary incontinence:**

Definition: The inability to hold urine in the bladder due to loss of voluntary control over the urinary sphincters resulting in the involuntary passage of urine is defined as urinary incontinence. A continent child thus must be dry at all times and must void spontaneously. Those who are on CIC and remain dry are termed as pseudocontinent. 

**Answer 2 **

Continence mechanism for feces includes several factors such as –

1. Intact anal sphincters
2. Anorectal sensation
3. Rectal compliance
4. Colon transit time/motility
5. Stool volume and consistency
6. Adequate cognitive function
7. Appropriate bathroom facilities
8. Position of defecation (squatting or sitting to facilitate the straightening of anorectal angle)

The structural and functional integrity of anorectal unit which is composed of first 4 factors is the key to fecal continence, of which normal anal sphincter function – both the external and internal anal sphincter - are critical parts of continence. (Fig. 1)

**Figure:**
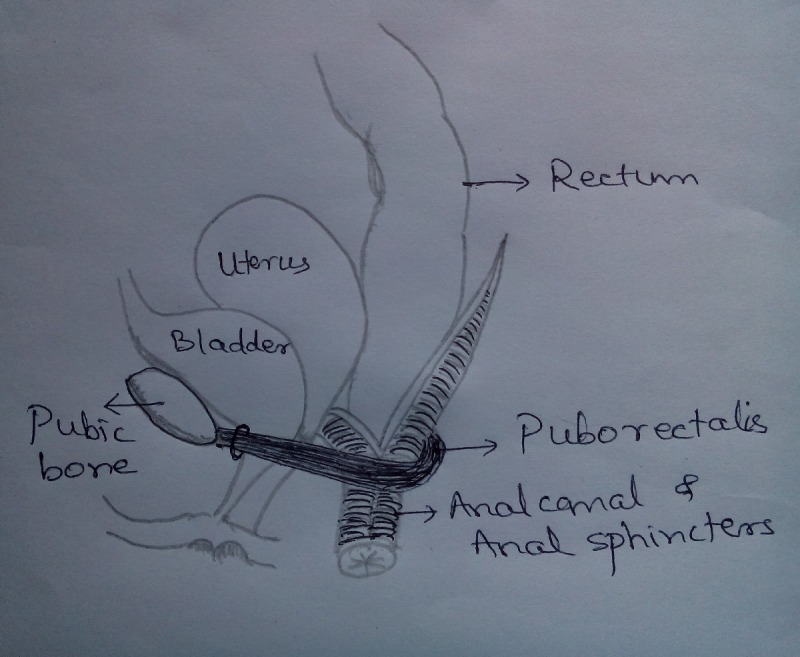
Figure 1: Sketch of anal sphincters.

Normal colonic motility propels stools in the rectum. Distension of rectum causes rectal contraction and pelvic floor and internal anal sphincter relaxation for defecation. If conditions are suitable, external anal sphincter relaxation occurs voluntarily causing defecation process to be completed. A normal sensory innervation at all levels, i.e. spinal cord, brain stem, thalamus and cortex is mandatory for the normal defecation process to occur and hence those children with sacral spinal abnormalities could have a neurological cause of fecal incontinence wherein they are unable to appreciate the fecal consistency, differentiate the sense of feces from rectal gas, quantity of feces, and co-ordination with other actions of perineal and abdominal muscles. 

The clinical parameters of the child with anorectal malformations can predict and prognosticate the long-term outcome of these children which is tabulated in Table 2 and Table 3.

**Figure:**
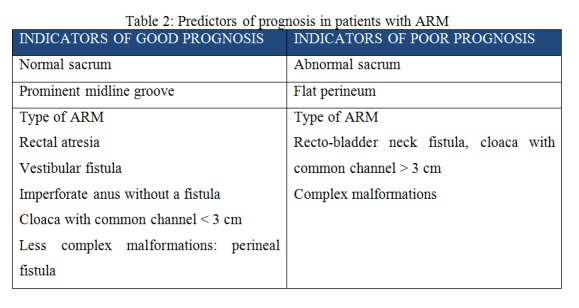
Table 2: Predictors of prognosis in patients with ARM

**Figure:**
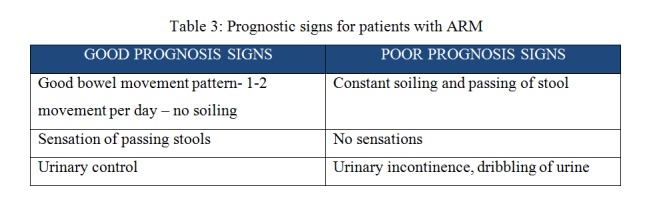
Table 3: Prognostic signs for patients with ARM

**Answer 3 **

Several scoring systems exist and the pediatric surgeon can choose any one scoring systems. Globally, there is still no consensus as to the best scoring system and also due to wide variations in extent of the anomaly and an inability to categorise the anomalies, the comparative evaluation is extremely difficult. Table 4 gives an overview of the existing scoring systems and the components assessed in these children. 

**Figure:**
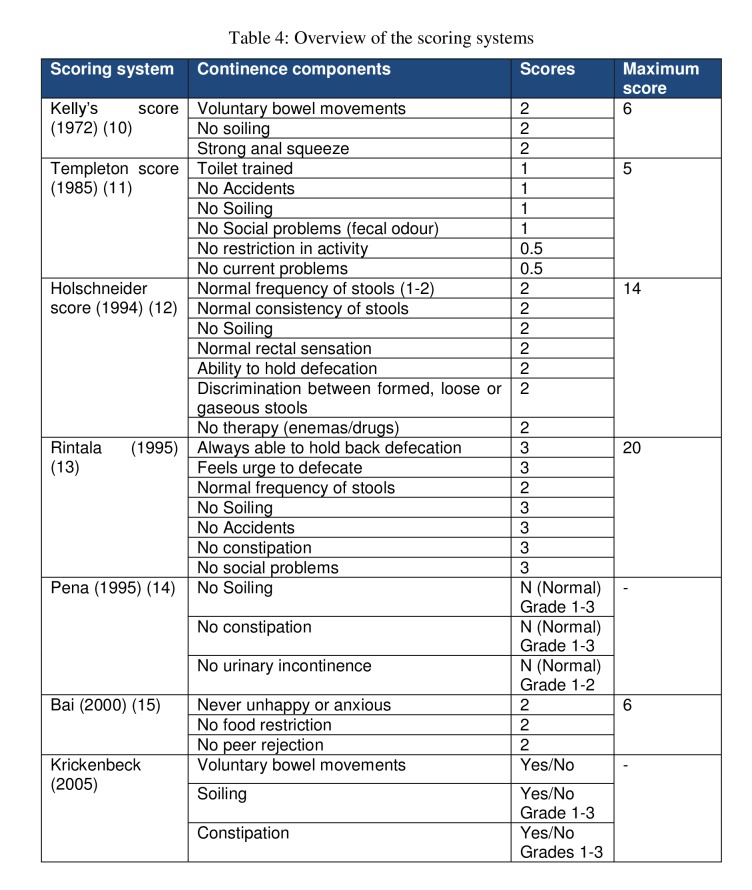
Table 4: Overview of the scoring systems

**Answer 4 **

Once a clinical evaluation is done and the severity of the fecal incontinence is assessed by utilizing the scoring system, further investigations are needed to ascertain the exact etiology of fecal incontinence. Depending on the cause of incontinence, treatment in the form of conservative or medical or surgical intervention is planned. Table 5 provides the sequence of diagnostic tests and the management thereof. 

**Figure:**
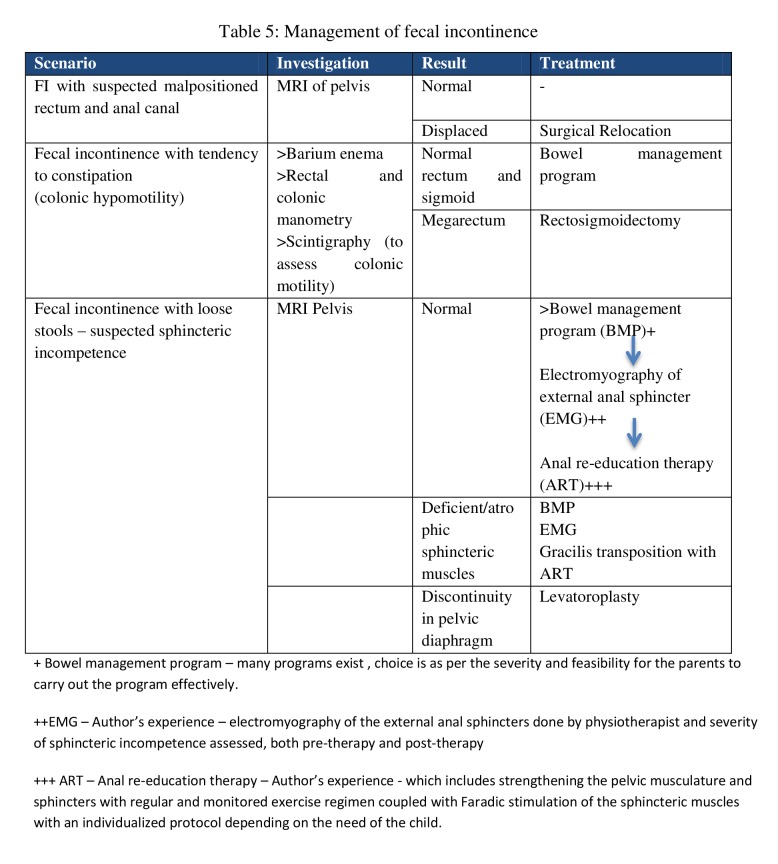
Table 5: Management of fecal incontinence

## Footnotes

**Source of Support:** Nil

**Conflict of Interest:** The author is editor of the journal. The manuscript is independently handled by other editors and she is not involved in decision making about the manuscript.

